# Early-life gut microbiota and its connection to metabolic health in children: Perspective on ecological drivers and need for quantitative approach

**DOI:** 10.1016/j.ebiom.2021.103475

**Published:** 2021-07-10

**Authors:** Ching Jian, Noora Carpén, Otto Helve, Willem M. de Vos, Katri Korpela, Anne Salonen

**Affiliations:** a Human Microbiome Research Program, Faculty of Medicine, University of Helsinki, Helsinki, Finland; b Children's Hospital, Pediatric Research Center, Helsinki University Hospital, University of Helsinki, Helsinki, Finland; c Finnish Institute for Health and Welfare, Department of Health Security, Helsinki, Finland; d Laboratory of Microbiology, Wageningen University & Research, Wageningen, Netherlands

## Abstract

The colonisation and development of the gut microbiota has been implicated in paediatric metabolic disorders via its powerful effect on host metabolic and immune homeostasis. Here we summarise the evidence from human studies on the early gut microbiota and paediatric overweight and obesity. Manipulation of the early gut microbiota may represent a promising target for countering the burgeoning metabolic disorders in the paediatric population, provided the assembly patterns of microbiota and their health consequences can be decoded. Therefore, in this review, we pay particular attention to the important ecological drivers affecting the community dynamics of the early gut microbiota. We then discuss the knowledge gaps in commonly studied exposures linking the gut microbiota to metabolic disorders, especially regarding maternal factors and antibiotic use. This review also attempts to give directions for future studies aiming to identify predictive and corrective measures for paediatric metabolic disorders based on the gut microbiota.

Gut microbiota; Metabolism; Paediatric overweight and obesity; Ecological driver; Dynamics; Infants

## 1 Introduction

The collection of bacteria, archaea, viruses, fungi, and eukarya residing in the human intestinal tract, known as the gut microbiota, represents one of the most significant features contributing to physiological inter-individual variability [1]. The developing gut microbiota is inextricably and interdependently involved in the concurrent maturation of the endocrine, immune and metabolic pathways during early life [2]. Considerable attention has been given to prevalent diseases at the interface between metabolism and immunity, including obesity, diabetes mellitus, and non-alcoholic fatty liver disease (NAFLD). While these diseases are manifested in later life, they are affected by metabolic programming occurring in early life and thus may be mitigated or averted via early correction of risk factors [3,4]. Accordingly, research in experimental models that demonstrates microbiota-dependent transmissibility of host disease phenotype provides some evidence that colonisation history, developmental trajectories, and disturbance in the early gut microbiota are implicated in metabolic and immune health later in life [5], [6], [7], [8]. To date, several prospective studies in humans have demonstrated links between deviations in the early life microbiota and paediatric overweight and obesity. Therefore, by understanding pivotal ecological drivers and environmental factors contributing to shifts in microbial equilibrium during the critical window of development, further deviations from the normal development may be forestalled. In this review, we discuss recent advances in understanding the connection between the early-life bacterial gut microbiota and metabolic health in children and adolescents, with a special focus on childhood obesity that has become a global public health crisis in the past decades [9].

## 2 Ecological perspective to the development of the human gut microbiota

**Fig. 1:**
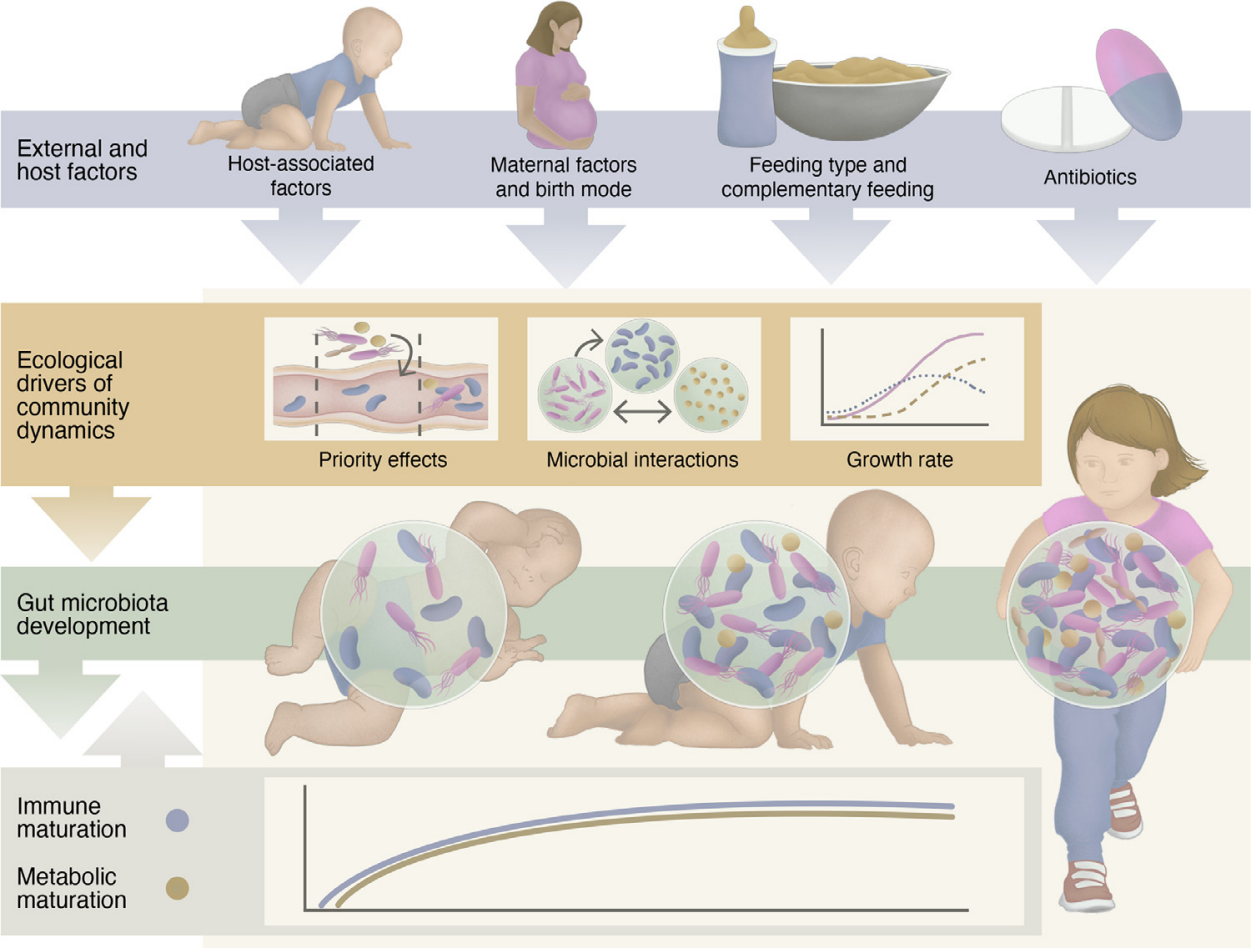
An overview of important host and external factors and ecological drivers affecting the infant's immune, metabolic and gut microbiota development.

It is generally recognised that the main events of microbial colonisation in infants occur during and after birth and include vertical (from the mother) and horizontal (from the immediate environment) transfers [10,11]. During the last decade, several descriptive sequencing studies reporting the potential existence of microbial communities in the placenta and amniotic fluid point to a possibility of *in utero* gut colonisation, although the available scientific evidence is insufficient to assert this hypothesis currently [11]. Colonisation is followed by relatively reproducible successional dynamics of the early microbiota that continues to mature into adolescence, as has been extensively reviewed previously [2,10,12]. Recent studies have shown that the maturation of the infant gut microbiota into an adult-like one is an intricate and continuous process involving phylogenetic [13] and functional [14] convergence. The convergence is governed by forces such as host diet and drugs, hormonal and metabolic status, genotype, and immune system that contribute to shaping the gut microbes into a highly individualised and relatively stable community. The true impact of potential stochastic forces [15] has not been reliably quantified, as most of the observed stochasticity is likely caused by unmeasured deterministic forces. The effects of individual external factors, such as birth mode, antibiotic use, and availability of breast milk on shaping the early gut microbiota are relatively well studied [16]. On the other hand, the combinatory (cumulative or counteractive) effects of these common exposures remain relatively unexplored. Similarly, the mechanisms by which the host regulates the gut microbial communities in infancy, such as secretions of mucus glycans, immunoglobulin A (IgA) and bile acids, are poorly understood. Moreover, little is known regarding the ecological drivers of early microbial community dynamics, including colonisation history [17], bacterial growth or replication rates [18], and interactions between the community members [19], which may equally have long-lasting health implications (Fig. 1).

A recent murine study demonstrated that the colonisation order of a seed community determines the resulting community composition, known as a priority effect. Priority effects may provide a link between certain microbial signatures observed in obese children and their early microbial exposures, such as decreased proportions of *Bifidobacterium* [20] that are often associated with caesarean (C-) section delivery, not being breastfed, and antibiotic use [16]. Priority effects partly dictate subsequent microbe-microbe interactions. For example, the lack of *Bifidobacterium* promotes the colonisation of streptococci [21], which have been linked to increased BMI in antibiotic-exposed infants [22]. On the other hand, the depletion of *Bifidobacterium* spp. could inhibit the colonisation of other bacteria key to infants’ growth due to the absence of trophic interactions [23].

Bacterial growth rate (the rate of change in the number of cells in a given habitat over time) represents an independent feature of the effect of the gut microbiota on the host and has been significantly correlated with several inflammatory and metabolic disorders in adults [18]. A higher bacterial replication rate as a measure of bacterial growth rate was observed before the diagnosis of necrotising enterocolitis in preterm infants [24]. Bacterial growth rates are highly relevant for gut bacteria that possess flexible metabolic capacity and short doubling times, such as many pro-inflammatory Proteobacteria that are initial colonisers and critical in education of host immunity [25]. Moreover, changes in the growth rates of particular bacteria e.g. catalytic [26] or keystone [27] species in response to environmental signals may herald the deviation of the gut microbiota development, leading to the collapse of normal gut communities and eventual dysbiosis-related disorders.

To study these ecological drivers that could provide understandings on disease aetiology and potential corrective approaches, it is necessary to quantify changes in the absolute abundance of biotic community members over time [19]. This has been largely overlooked in the preponderance of current microbiota studies based on a single snapshot of the developing microbiota, where sequencing-derived relative abundances of microbial taxa were analysed. On one hand, the use of relative abundance is problematic as its compositional nature (changes of abundances are mutually dependent due to a constant sum constraint) can mask true community dynamics [19] and lead to high false discovery rates [28]. On the other hand, absolute quantitation of the gut microbes provides richer insights into their immune and metabolic impact, as the magnitude of the immune responses or the concentrations of microbial metabolites largely depend on cell quantity [29]. Of note, growth rate measurement requires estimating cell density at a series of time points, which can be readily achieved by integrating absolute quantitation into widely used next-generation sequencing (NGS), termed quantitative microbiome profiling (QMP) [28,30]. QMP has been recently applied to understand health and disease in adults [30], but not yet in paediatric populations where the gut microbial interactions are highly dynamic. Importantly, no studies to date have comprehensively surveyed the changes of the gut microbes in absolute abundance during early development.

## 3 Impact of major perinatal factors on the development of early microbiota and metabolic health

### 3.1 Maternal obesity and diet

Maternal vaginal and faecal microbes represent the first and potentially the most numerous sources of bacteria the infant is exposed to. Since vaginal bacteria do not persistently colonise the infant gut [31], the maternal gut microbiota is the main source of colonisation in vaginally born infants [31,32]. The vaginal microbiota may yet have a substantial impact on the infant's early immunity considering the neonate's exposure to high quantities of bacteria during vaginal birth (up to 10^9^ bacteria per gram of vaginal secretions) [33], although this has been understudied.

Maternal obesity could introduce microbiota aberrations in the early-life microbiota relevant for metabolic health at least via two mechanisms. First, through the direct vertical microbiota transfer and priority effects, the maternal obesity-associated microbiota results in intergenerational effects [34,35] and possibly the development of metabolic disorders [35]. While dietary habits within the family as well as genetic and epigenetic factors are known to contribute to transgenerational obesity, maternal transmission of an altered gut microbiota has been attributed as one potential mechanism in offspring obesity and NAFLD in animal models [36]. The effect of maternal obesity on the infant's gut microbiota is less clear in humans [37], likely due to multiple confounding factors. Indeed, there are some data supporting the effect of obesity or metabolic disease on the maternal gut microbiota during pregnancy [38]. Interestingly, two studies reported that the gut microbiota in infants born to obese mothers had a reduced proportion of Proteobacteria [35,39] that are normally abundant during the first weeks of life [12]. Colonisation of germ-free mice with the gut microbes from the infants born to obese mothers yielded a persistently compromised innate immune cell function compared to controls, which was thought to exacerbate the development of NAFLD and excessive weight gain in the mice on an obesogenic diet [39]. Second, maternal overweight and obesity is a main risk factor for C-section delivery [40], which alters the early microbiota development as discussed in the next section. C-section delivered infants of overweight mothers were reported to have a higher risk of overweight at one year compared to vaginally delivered infants [35], stressing the importance of microbial colonisation of the newborn and priority effects in determining later microbiota and host development.

Maternal diet during the periconceptional period is one of the critical and modifiable factors on child health [41], effects of which could be partly driven by the gut microbiota. In mice, gut microbiota–derived metabolites represented by short-chain fatty acids have been shown to reach the embryos via the maternal liver and blood stream, shaping embryonic energy metabolism and subsequently postnatal energy homeostasis [42]. Garcia-Mantrana et al. reported that the maternal microbiota associated with a specific maternal dietary pattern (lower intakes of fibre and n-3 fatty acids, and higher intakes of animal protein and saturated fats) along with other perinatal factors shaped the neonatal gut microbiota that may predispose infants to overweight during the first 18 months of life [43]. A recent study found that maternal consumption of artificially sweetened beverages during pregnancy was associated with the depletion of *Bacteroides* spp. in the infant gut microbiota as well as elevated levels of urine succinate that correlated with infant BMI [44]. Exposure to maternal high-fat diet, associated with increased intrahepatic triglyceride levels in the offspring in experimental models [36], altered the neonatal gut microbiota in humans [45]. Short-term high-fat diet (saturated fats specifically), possibly through increases in bile acid secretion [46], induces specific compositional changes in the gut microbiota, such as increased pro-inflammatory *Bilophila*/Proteobacteria that are associated with liver steatosis [47] and hence may contribute to the intergenerational transmission of NAFLD and obese phenotypes.

To date, most dietary interventions in women at child-bearing age aiming to improve maternal health have not accounted for the diet-induced changes in the maternal gut microbiota that could positively or negatively influence metabolic outcomes in the children. For example, a low-energy diet used for pre-conception weight loss [48] is frequently linked to reduced *Bifidobacterium* [49] due to the insufficient amount of dietary fibre and starch. Whether the reduced abundance of *Bifidobacterium* would imprint in neonates’ gut microbiota and mediate potential metabolic consequences later in life is unknown. With the burgeoning trend of alternative diet regimes, such as ketogenic diets, future studies are needed to investigate their impact on the maternal and neonatal gut microbiota in relation to intergenerational transmission of metabolic outcomes.

### 3.2 Birth mode

Mode of delivery is one of the foremost contributors to sculpturing the infant's microbiota. The first bacteria populating the gut of naturally born infants come primarily from the mother via faecal-oral route [31,32]. Since the first strain-resolved metagenomic studies documenting the disruption of mother-to-neonate microbiota transmission in infants born by C-section [50], the more recent studies have also addressed the functional implications on the community and strain levels [51], [52], [53]. In C-section born infants, bacteria from the hospital environment colonise the gut and gradually become replaced by bacteria better adapted to the intestinal environment [10,52,53]. As a result, the assembly and dynamics of intestinal bacteria in C-section babies differ appreciably from those in the vaginally born, typified by the lack or decrease of Bacteroidetes, the decrease of bifidobacteria and respective increases in Proteobacteria [10]. Some studies have reported this imprint to last up to 18 months [54].

C-section delivery has been implicated in various immune disorders [55] as well as associated with an increased risk of offspring obesity in two earlier meta-analyses of 24 studies [56,57], which may be mediated by delayed maturation of the gut microbiota [58,59]. Nevertheless, some recent studies have found null associations between C-section and offspring corpulence, citing that the earlier studies failed to account for maternal BMI and prenatal factors [60].

Subtle differences in the infants born by elective C-section (no labour) versus emergency C-section (labour) have been observed in some studies [61], which are attributable to the lack of immune responses within the uterine cavity normally induced by labour [62]. It remains unknown whether these differences are pertinent to the different risks of childhood overweight and obesity between elective and emergency C-section [63,64].

### 3.3 Feeding type

After birth, breastfeeding becomes one of the most important and modifiable determinants of infant gut microbial colonisation. The differences in the gut microbiota composition between breastfed and formula-fed infants are well-established [65]. Human milk is a natural modifier of gut microbial composition via the prebiotic properties of human milk oligosaccharides (HMOs), its immunoglobulins and antimicrobial compounds that select for certain bacteria, and acting as a potential source of microbial inoculum that may play a role in infant growth [66]. The selective capacity for utilisation of HMOs favours the growth of *Bifidobacteriaceae* in the breastfed infant's gut [67]. The HMO composition in breast milk has been associated with infant growth and body composition [68,69], and theorised to contribute to the protective effects of breastfeeding against childhood overweight and obesity [70] and type 2 diabetes [71] via the gut microbiota [72]. Breastfeeding has been shown to partially restore the disruptions in the infant's gut microbiota caused by C-section, specifically the depletion of bifidobacteria, potentially mediated by the abundant amount of α1-2 fucosylated oligosaccharides in the breast milk of mothers with a functional FUT2 allele [73,74].

In contrast, a study in 1087 infants suggested that formula feeding stimulated changes in the gut microbiota (higher microbiota diversity and increased relative abundance of *Lachnospiraceae*) that partially explained the increased risk of overweight at one year [75]. A meta-analysis including seven studies across three continents reported that the gut microbial functions in non-exclusively breastfed neonates were characterised by increased carbohydrate metabolism and reduced lipid metabolism/homeostasis as well as decreases in detoxification pathways [76]. Early complementary feeding is a risk factor for childhood obesity [77] and NAFLD in adolescents [78], and has been linked to the altered abundances of *Roseburia* and *Bilophila wasworthia* [79].

### 3.4 Antibiotics

Early-life antibiotic exposure is well-known for disrupting gut microbiota homeostasis, giving rise to elevated proportions of Proteobacteria and reduced *Bifidobacterium* in the infant's gut microbiota [80,81]. Antibiotic use in infancy increases the risk of childhood overweight and obesity depending on gender and exposure timing [82]. In a large Finnish cohort of healthy children, antibiotic exposure before six months of age, or repeatedly during infancy, was associated with increased body mass at the age of 24 months [83]. There is some evidence suggesting that antibiotics alter host metabolism by reshaping bile acid profiles [80,84].

A single course of amoxicillin, the most commonly used antibiotic in neonates, profoundly biased the maturation of the early gut microbiota in two- to three-month-old infants toward low abundance of bifidobacteria and increased abundance of clostridia [81]. In older children (two- to seven-year-olds), exposure to macrolides was shown to profoundly after the gut microbiota, specifically reducing bile-salt hydrolase producing bacteria including bifidobacteria. The changes were linked to increased BMI potentially via altered bile acid metabolism [80]. Uzan-Yulzari et al. recently reported that neonatal antibiotic exposure was associated with reduced growth during the first six years of life particularly in boys, whereas antibiotic use after the neonatal period was associated with excessive childhood weight gain in both genders. These effects were likely mediated by the gut microbiota, specifically the decreased abundance and diversity of bifidobacteria, which remained detectable even 24 months after exposure [85]. Alarmingly, antibiotic exposure during the breastfeeding period may eliminate the beneficial metabolic effects of breastfeeding by altering the gut microbiota [86].

Antibiotics are among the most commonly prescribed medications to newborns and children. The first exposure may come through the umbilical cord following intrapartum antibiotic prophylaxis (IAP), the preventive antibiotics given to the vaginally delivering mothers who screen positive for group B streptococcus (GBS) as well as during C-section or assisted vaginal delivery. IAP administration has been linked to an increased risk of obesity and immune diseases in the child [10]. The gut microbiota composition of IAP-exposed infants appears to be similar to that of C-section-delivered infants [10], which nevertheless could have been confounded by several factors as the few available studies are of small sample size and high heterogeneity in study design [87]. IAP exposure, particularly to multiple antibiotic classes, led to year-long alterations in the development trajectory of the gut microbiota in vaginally born infants, suggesting the importance of antibiotic class and selectivity in reshaping the gut microbiota [88].

Antibiotic prescribing patterns in infants and childhood are extremely variable, likely emanating from differences in the physicians’ clinical experiences to some extent [89]. For instance, the duration of parenteral antibiotics for infants with bacteremic urinary tract infection varied from 1 to 24 days depending on the treatment location of the patient [90]. Recent studies have shown possible dose-response [91] and class-specific [92] effects of the antibiotic exposure on children's growth trajectories. There is, however, a general paucity of research exploring the impact of antibiotic course, class and timing of administration on the neonate's gut microbiota in relation to health consequences.

### 3.5 Associations between early-life microbiota and adiposity in later infancy

While early disturbances on the microbiota seem to be transient and largely return to normal after weaning [93], temporary perturbations in the ecosystem during the sensitive developmental period may still increase the propensity to later metabolic dysregulation as demonstrated in experimental models [6]. Ten prospective studies have examined the link between the early-life gut microbiota and subsequent development of adiposity in the same subjects (Table 1). These studies, albeit differences in methodologies and confounding factors, have shown a general trend where higher relative abundances or the presence of *Bacteroides* spp. and lower relative abundances of bifidobacteria in early infancy are associated with paediatric overweight and obesity. *Staphylococcus* spp. may also be predictive of childhood BMI, although the directionality of the association is inconsistent [94,95]. Interestingly, microbiota features have been reported to associate with child adiposity as early as in the meconium i.e. the earliest stool [96]. While these findings imply that changes in the gut microbiota are detectable much earlier than the manifestation of obesity, prospective long-term cohort studies as well as randomised controlled trials (when ethical) are needed to ultimately determine whether the microbiota features in infancy are a mere reflection of, or a casual contributor to the pathogenesis of childhood overweight and obesity. This is further complicated by the fact that the gut bacteria undergo rapid undulations during the first year of life, sensitive to multiple sources of perturbation that cannot be captured by one snapshot as done in most existing studies. Moreover, none of the abovementioned studies employed metagenomic sequencing that could unravel specific bacterial species, strains and functions as well as potential viruses or fungi involved in the development of paediatric obesity. As taxonomic units have inherent limitations in resolution, future studies employing metagenomic analysis may benefit from genome-based analysis frameworks, where individual genomes present in a sample are directly taxonomically and/or functionally annotated [97,98].

**Table 1:** Summary of studies on associations between early-life gut microbiota and infant weight development or childhood overweight and obesity.

Study	Country	Sample size	Age at collection of faecal sampling (d=day; w=week; m=month)	Age at assessment of adiposity (final time point; y=year)	Key findings on the gut microbiota	Potential confounders	References (PMID)
Kalliomäki et al.	Finland	49	6m-12m	7y	Increased bifidobacteria in children remaining normal weight; increased *Staphylococcus aureus* in children developing overweight.	Maternal BMI, IAP, CS type	18326589
Vael et al.	Belgium	138	3w-52w	3y	Increased *Bacteroides fragilis* and decreased *Staphylococcus* associated with obesity later in childhood.	IAP	21605455
Luoto et al.	Finland	30	3m	10y	Increased bifidobacteria in children remaining normal weight (non-significant; P=0.087).	IAP, CS type	21150648
White et al.	Norway	218	4d-120d	0•5y	Developmental trajectories of *Staphylococcus* species and *Escherichia coli* associated with expected growth; presence of *Bacteroides* spp. at one month associated with growth.	-	23671411
Scheepers et al.	The Netherlands	909	1m	10y	Presence of *Bacteroides fragilis* group at one month associated with a higher BMI later in childhood.	-	25298274
Dogra et al.	Singapore	75	6m	1•5y	Infants who acquired a profile high in *Bifidobacterium* and *Collinsella*, and low in *Enterobacteriaceae* at a later age associated with lower adiposity at 18 months of age.	Ethnicity, antibiotics, CS type	25650398
Korpela et al.	The Netherlands & Finland	162	3m	5-6y	At three months of age, *Bifidobaterium* spp. negatively and streptococci positively associated with BMI at five to six years.	-	28253911
Forbes et al.	Canada	1087	3m-4m	1y	Longitudinal associations between the composition of gut microbiota at three to four months of age and weight status at one year of age.	-	29868719
Stanislawski et al.	Norway	165	10d-730d	12y	Over 50% of the variation in obesity at 12 years of age explained by the pattern of types of gut microbiota at two years of age.	CS type	30352933
Korpela et al.	Finland	212	<1d	3y	Increased *Bacteroides fragilis* in the meconium associated with overweight at the age of three years.	Maternal BMI, CS type	32638554

BMI: body mass index; IAP: intrapartum antibiotic prophylaxis; CS: caesarean section.

## 4 Potential mechanisms linking the early-life microbiota to metabolic dysregulation

**Fig. 2:**
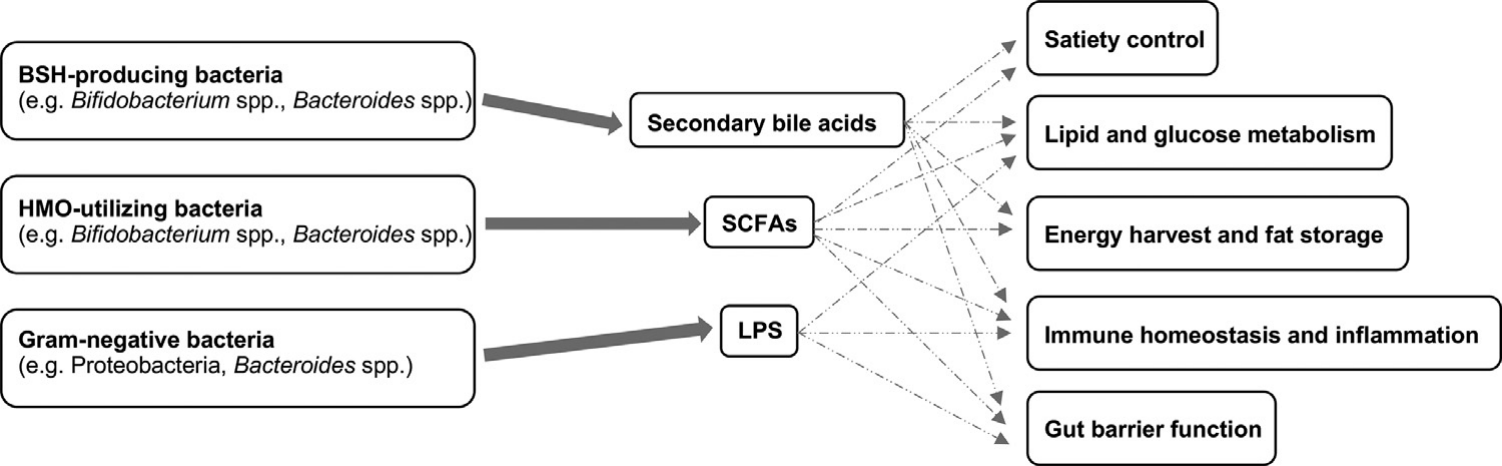
Key gut bacteria, bacterial metabolites and mechanisms potentially involved in the development of paediatric metabolic disorders. BSH: bile salt hydrolase; HMO: human milk oligosaccharide; SCFAs: short-chain fatty acids; LPS: lipopolysaccharide.

Evidence from previous studies, mainly in experimental models, suggests that the gut microbiota contributes to metabolic diseases via several mechanisms, including increased energy harvest and fat storage, regulation of lipid and glucose metabolism, gut barrier function, induction of low-grade inflammation, and to a lesser extent satiety control mediated by gut hormones as well as interactions with host genetics [99]. These mechanisms, chiefly ascribed to the actions of lipopolysaccharide (LPS), short-chain fatty acids (SCFAs) and bile acids, may work in parallel or synergistically to alter physiology in a developing newborn (Fig. 2). Additionally, recent studies have identified other microbiota-dependent metabolites that play a role in metabolic diseases mediated by pro-inflammatory pathways, such as the microbially produced histidine metabolite, imidazole propionate [100], and the microbial metabolite succinate [101].

During the critical time window in neonatal life, immune development is reliant on triggers provided by the neonatal microbiota as well as the maternal microbiota [102]. Maturation of innate immunity in return creates a selective pressure for the gut microbes, impelling their temporal development. The initially colonising Gram-negatives, mainly Proteobacteria and *Bacteroides* spp. are believed to play a role in this process via their structural component LPS, one of the best studied pathogen-associated molecular patterns (PAMPs) that represent targets of innate immune recognition [25]. A previous study demonstrated that vaginally-delivered mice acquired resistance to LPS shortly after birth, leading to immunotolerance [103]. While exposure to LPS during early postnatal development is required to educate the immune system, the sustained exposure to LPS with compromised gut barrier function results in the activation of pro-inflammatory pathways and subsequent metabolic dysregulation [104]. The relative abundances of Proteobacteria species are initially high and gradually decrease over time in full-term vaginally born neonates, which can be partly ascribed to the selective suppression of HMOs and secretory IgA in the human milk [105]. Neonatal antibiotic exposure, including IAP and postnatal antibiotic administration, has been associated with an increase in LPS-producing Proteobacteria [106]. The bacterial LPS structures between bacterial genera (e.g. between *Escherichia* and *Bacteroides* spp.) and even species can substantially differ in their inflammatory potential [107] and one study suggests that early colonization with the former may be needed for appropriate immune activation and endotoxin tolerance that decrease susceptibility to immune diseases [25]. Hence, the exposure to immunogenic LPS at the appropriate timing in early life may be part of normal development rather than harmful. However, the actual role of different LPS-producing bacteria in the early education of immunity as well as development of childhood obesity is currently unclear. It also remains unknown whether other PAMPs have a role in the pathophysiology of excessive weight gain in early life.

SCFAs (acetate, propionate and butyrate) are the primary microbial metabolites of HMO fermentation that contribute to energy homeostasis, modulate host adiposity, regulate immune functions, and alter gene expression of host satiety hormones [108]. *Bifidobacteria spp.* (producing acetate and lactate that can be converted into butyrate by other colonic bacteria), consistently enriched in vaginally born breast-fed infants, are major HMO utilisers that have been given credit for improved gut and systemic health in infants. The multifunctional effects of *Bifidobacterium* spp. include the amelioration of diet-induced endotoxemia and inflammation in the gut, liver and adipose tissue, and the deconjugation of bile acids influential to the host's lipid metabolism and energy expenditure [109,110]. *Bifidobacterium infantis,* a key early coloniser, has also been reported to directly correlate with the amount of secreted IgA antibodies [111], which are the centrepiece of protective humoral mucosal immunity. Therefore, the depletion of *Bifidobacterium* spp. may promote loss of immune- and metabolic homeostasis in infants and further, diseased states. Of note, a meta-analysis found a loss of bifidobacteria in healthy breastfed infants, indicated by increased faecal pH, over the past century (1926-2017) [112]. Similarly, a recent metagenomic study on the contemporary American infant gut microbiome reported reduced capacity for HMO utilisation and other ecosystem services provided by the gut microbiota to the host [113]. Interestingly, reduced levels of HMO-metabolising genes and the main *Bifidobacterium* species encoding them were not related to infant diet, alluding to the role of longer-term, potentially even generational influences on this early dysbiosis. Finally, as mentioned earlier, the scarcity of *Bacteroides spp*., the main propionate producers in the human gut, is a taxonomic signature of C-section born infants. While several mechanisms have been identified regarding how propionate can promote metabolic health [114], for now, very little is known about the physiological implications of reduced early colonisation by *Bacteroides* spp.

Bile acid metabolism is critical for the absorption of dietary fat and plays an important signalling role in metabolic regulation via the nuclear receptor FXR and the G-protein-coupled bile acid receptor-1, especially in the gut-to-liver axis [115]. Intestinal bacteria deconjugate primary conjugated bile acids by bile-salt hydrolase and modify them into secondary and tertiary forms [116]. Accordingly, the bile acid composition is largely shaped by the gut microbiota, and has been suggested to contribute to obesity susceptibility in both humans and mice [117]. On the other hand, bile acids pose a selective pressure on the gut microbes due to their toxicity for bacterial cells [116], rendering them influential for the early gut microbiota maturation based on a recent study in mice [118]. A recent longitudinal study suggested that neonates experience a drastic transition of the bile acid profiles during lactation that coincides with the transition of the microbiota profiles [119]. Perturbations of bile acid and/or microbiota profiles, such as antibiotic-driven depletion of bile-salt hydrolase producing bacteria [80], during this critical period of time may therefore have negative metabolic consequences. Currently, no studies have investigated the interactions between bile acids, the gut microbiota and host adiposity simultaneously in children.

## 5 Modelling gut microbiota dynamics in relation to metabolic health

Prediction and early intervention of excessive weight development and related metabolic dysregulation is the cornerstone of preventive healthcare to curb the epidemic of obesity. While changing dietary patterns and lifestyle of children is beneficial, another mode of intervention is reducing factors that increase the risk of diet-induced metabolic dysfunction. In this regard, the gut microbiota represents a promising target. A few studies have developed prognostic prediction models using known risk factors for childhood obesity e.g. maternal pre-pregnancy BMI and birth weight, achieving varying performances [120]. Recent studies employing machine learning algorithms have suggested comparable predictive values of the gut microbiota in childhood obesity, though external validation is lacking [96,121].

Reliable predictive models applied to dynamic systems, such as growing infants or developing gut microbiota, should have the ability to absorb changes in the environments upon their introduction. In terms of the gut microbiota, this requires a better understanding of the community dynamics of the gut microbiota, such as priority effects, intra- and interspecies interactions, and the consequences of alternative assembly patterns. Various mathematical models have been applied to predict the dynamic behaviours of the gut microbiota. For instance, the generalised Lotka-Volterra (gLV) model, which describes the change over time of a population of microbial members as a function of their intrinsic growth rates and pairwise interactions (e.g. predator-prey or mutualism), has become one of the most popular models [122]. Nevertheless, these simplistic pairwise interactions are insufficient to capture complex interaction networks occurring in a microbial community [123], calling into question the utility of the gLV model. To capture non-pairwise interactions mediated by environmental factors e.g. antibiotic perturbation, time series modeling from multiple sampling is essential [124]. Genome-scale metabolic models (GEMs) computationally describe gene-protein-reaction associations for entire metabolic genes in an organism [98] and have been utilised to accurately predict growth trajectories and changes in body compositions up to age six months in healthy neonates [125], which could be integrated with GEMs of the gut microbiota that contributes to a significant portion of daily metabolic fluxes. In addition, GEMs could prove a functional perspective with biologically relevant resolution taxonomically and prediction of metabolic interactions between microbial taxa in metagenomic studies, potentially revealing the “driver” microbes that contribute to the development of metabolic diseases.

## 6 Therapeutic potential of the early-life gut microbiota in paediatric metabolic disorders

The gut microbiota in infants is often regarded as having high plasticity due to its low diversity and rapid development, in comparison to the microbiota in adults, and has become an attractive target for modifications aiming to improve health in later life [126]. Microbiota-targeted modifications can be broadly categorised into three groups: depletion (e.g. antibiotics), modulation (e.g. pre- and probiotics or dietary intervention) and replacement (e.g. faecal microbiota transplantation (FMT)).

The growth promoting effect of antibiotics has been long appreciated in animal production [127] and exploited to increase growth rates in malnourished children in low-income countries [128]. Since specific antibiotic classes are highly selective against particular microbes, this property could be leveraged to manipulate their collateral effect on the microbiota in the future [129].

Probiotics have increasingly been administered to infants and children in recent years, and shown a larger impact on C-section-delivered infants compared to their vaginally born counterparts [130]. However, mixed results have been documented from few trials examining the effect of early administration of probiotics on excessive weight development in neonates. The perinatal use of *Lactobacillus rhamnosus* appeared to modify children's growth patterns by inhibiting excessive weight gain during the first few years of life [131], while other studies in 179 healthy, term infants using *L. paracasei* subsp. paracasei F19 found no short-term [132] and long-term [133] metabolic benefits. The disparities between the studies may be partially explained by priority effects arising from different probiotic strains administered at different time points and for different durations. It is also worth mentioning that there is insufficient evidence supporting the role of *Lactobacillus* spp., while being the most commonly used probiotic strains, in weight control in the infant age group, especially considering their low abundance and strain-specific effects [134]. Thus, hypothesis-driven probiotic interventions using the strains known to be involved in energy metabolism are urgently needed.

Replacement of the gut microbiota by FMT represents the most powerful option to re-direct the microbiota. A recent FMT trial in adolescents with obesity led to a slight but significant reduction in visceral adiposity [135]. A recent proof-of-concept study found that maternal FMT, not vaginal swabbing, corrected the C-section associated depletion in *Bacteroides* spp. and the delayed maturation of *Bifidobacterium* in neonates [31]. While the metabolic consequences in the treated infants are to be followed, this study highlights the importance of the vertical microbiota transfer on the colonisation.

## 7 Conclusion

Over the past decade, research in animal models and epidemiological research in humans has provided evidence linking the disruption of multifaceted microbiota-host dialogues in infancy to negative metabolic and immune health later in life. To move beyond associations, hypothesis-directed interventions in children during development are the necessary next step toward tapping into the therapeutic potential of the gut microbiota in paediatric metabolic disorders. Importantly, ecological drivers of early microbial community dynamics could provide new insight into immune and metabolic homeostasis as well as susceptibility to inflammatory diseases later in life. Therefore, we posit a need for novel approaches that enable studying host-microbe dynamics in a community ecology perspective, which can be utilised for devising effective diagnostic and therapeutic strategies.

## 8 Outstanding questions

While previous studies have generated ample evidence on the importance of the gut microbiota in the critical window of development, it remains unclear whether alterations in the gut microbiota occurring early in time would exert the same impact on the host as those lasting longer or arising later, which could vary across different metabolic outcomes as well. Therefore, longitudinal analysis of gut microbiota, host faecal and serum biomarkers and detailed background data are of paramount importance in understanding the time window of microbiota effects on host metabolism, which determines the timing of administrating corrective measures, as well as in inferring mechanisms. Based on this knowledge, hypothesis-driven interventions in children should be derived and longitudinal experiments designed as the ultimate way to establish causality.

There is currently scant information on the mechanisms of maternal effects, such as the role of the microbiota in vertically transmitted metabolic phenotypes, and the effects of maternal medication and nutrition on neonates during the periconceptional period and during lactation. These are imperative questions to answer, considering prenatal preventative interventions are required to break the cyclical process of intergenerational metabolic dysregulation.

## 9 Search and selection criteria

This review is based on a systematic search in PubMed and MEDLINE using the terms (microbiota OR microbiome) AND (metabolic health OR metabolism OR obesity OR overweight) as of February 10^th^, 2021. We limited our search to articles on full-term infants and written in English in the last 15 years.

## Contributors

CJ conceptualised, conducted the literature search, designed figures and tables and wrote the draft with important inputs from KK, WMdV and AS. KK and AS provided critical feedback and revised the draft. NC conducted the literature search with the supervision from OH. OH provided resources and project administration. All authors have read and approved the final version of the manuscript. Any pharmaceutical or other company was not involved in any work related to writing of this article.

## Declaration of Competing Interest

None.
